# Circulating MicroRNA Profiling for Phenotypic Stratification in Patients with Metabolic Dysfunction-Associated Fatty Liver Disease: A Candidate-Based Study

**DOI:** 10.3390/cimb48030272

**Published:** 2026-03-04

**Authors:** Sumbal Nida, Dilshad Ahmed Khan, Muhammad Amjad Pervez, Nayyar Chaudhry, Mohammad Qaiser Alam Khan, Alveena Younas

**Affiliations:** Armed Forces Institute of Pathology, National University of Medical Sciences, Rawalpindi 46000, Pakistan; docsumbalnida@gmail.com (S.N.); dramjad14@gmail.com (M.A.P.); nayyarchaudhry@yahoo.com (N.C.); mqak68@gmail.com (M.Q.A.K.); dralveena.younas4@gmail.com (A.Y.)

**Keywords:** metabolic dysfunction-associated fatty liver disease, MAFLD phenotypes, microRNA, diagnostic accuracy, type 2 diabetes, obesity, insulin resistance, metabolic risk factors

## Abstract

Metabolic dysfunction-associated fatty liver disease (MAFLD) comprises phenotypic subgroups, including type-2 diabetes-associated MAFLD (T2D-MAFLD), obesity-associated MAFLD (OB-MAFLD), and lean MAFLD (L-MAFLD). Emerging evidence indicates that dysregulation of miRNAs plays a key role in MAFLD pathogenesis and progression. This study evaluated the diagnostic accuracy of a plasma miRNA-based signature as a non-invasive biomarker for early detection and phenotypic stratification of MAFLD. A total of 393 MAFLD patients and 109 healthy controls were enrolled. Plasma expression of miR-122, miR-103a, miR-222, miR-15a, miR-34a, miR-192, miR-197, and miR-99a was quantified using Reverse transcription polymerase chain reaction. Compared to controls, MAFLD patients exhibited significant upregulation of miR-122, miR-103a, miR-222, miR-15a, and miR-34a, alongside downregulation of miR-197 and miR-99a. Multinomial logistic regression revealed phenotype-specific associations: miR-103a, miR-34a, and miR-197 with T2D-MAFLD; miR-122, miR-222, and miR-99a with OB-MAFLD; and miR-15a with L-MAFLD. Receiver operating characteristic analysis demonstrated highest individual diagnostic accuracy for miR-197 in T2D-MAFLD (AUC = 0.784), miR-99a in OB-MAFLD (AUC = 0.869), and miR-15a in L-MAFLD (AUC = 0.776). Integrating combined miRNA panels with biochemical markers further improved diagnostic performance and clinical utility, achieving high positive and negative predictive values. In conclusion, plasma miRNA signatures enable phenotype-specific discrimination of MAFLD subtypes and may serve as promising non-invasive tools pending multi-center validation.

## 1. Introduction

Metabolic dysfunction-associated fatty liver disease (MAFLD) is a recently endorsed term that more accurately reflects the metabolic etiology of hepatic steatosis. It encompasses three clinically distinct phenotypes—type 2 diabetes-associated MAFLD (T2D-MAFLD), obesity-associated MAFLD (OB-MAFLD), and lean MAFLD (L-MAFLD)—each defined by underlying metabolic risk factors and displaying a varied spectrum of disease progression, fibrosis risk, and therapeutic response [1]. T2D-MAFLD is often linked to rapid progression toward fibrosis and hepatocellular carcinoma, whereas OB-MAFLD is highly prevalent, particularly among individuals with central adiposity. L-MAFLD, though less common, exhibits comparable hepatic and systemic risks despite a normal body mass index (BMI) [2].

MAFLD imposes a substantial burden in the Southeast Asian population. Regional meta-analysis estimate a pooled prevalence of approximately 34% (23–47%), reflecting rapid urbanization, lifestyle transitions, and genetic predisposition. Importantly, a recent meta-analysis from Pakistan revealed a pooled NAFLD prevalence of 29.8% (95% CI 21.4–39.0%) in the general population. The prevalence increases markedly among high-risk groups, including individuals with diabetes (58.5%), hypertension (74.1%), and obesity (47.4%), underscoring the strong association between metabolic risk factors and hepatic steatosis in these populations. These findings highlight the need for region-specific molecular characterization of phenotype-specific early diagnostic strategies to address the growing MAFLD burden in Pakistani populations [3].

MicroRNAs (miRNAs) are small non-coding RNAs generated through a tightly regulated biogenesis pathway involving Drosha- and Dicer-mediated processing of primary transcripts. Mature miRNAs regulate gene expression post-transcriptionally by binding to complementary sequences in target mRNAs, resulting in translational repression or degradation of mRNA. Notably, circulating miRNAs exhibit remarkable stability in tissues and body fluids due to encapsulation within exosomes or association with RNA-binding proteins, rendering them attractive non-invasive diagnostic biomarkers [4]. miRNAs have been investigated to reflect hepatocellular injury, metabolic dysregulation, inflammation, and fibrosis, frequently preceding clinical or biochemical abnormalities in patients with MAFLD. This characteristic makes them ideal for early diagnosis, risk stratification, and phenotypic differentiation in MAFLD, a condition that currently lacks phenotype-specific diagnostic tools.

A growing body of evidence implicates specific miRNAs in the development and progression of MAFLD through regulation of lipid metabolism, insulin resistance, inflammation, and fibrosis. Specifically, miRNA-122-5p (miR-122) regulates lipid synthesis in hepatocytes by modulating sterol regulatory element-binding protein-1c (SREBP1c), acetyl-CoA carboxylase (ACC), and fatty acid synthase (FASN). Its expression is elevated during hepatocellular injury and correlates with steatosis severity [5,6]. miRNA-222-3p (miR-222) influences lipid oxidation and adipocyte differentiation and is associated with obesity-related metabolic dysfunction [7,8,9], while miRNA-103a-3p (miR-103a) promotes hepatic insulin resistance by targeting caveolin-1 (CAV-1) and is strongly linked to diabetes pathogenesis [10]. miRNA-15a-5p (miRNA-15a) modulates insulin signaling and exhibits increased expression in lean individuals with metabolic dysfunction [11,12]. Furthermore, miRNAs contribute to disease progression through effects on apoptosis and fibrosis; miR-34a-5p (miR-34a) induces hepatocyte apoptosis and is elevated in advanced MAFLD, correlating with cytokeratin-18 (CK-18) and alanine aminotransferase (ALT) [13,14]. miR-192-5p (miR-192) is associated with steatosis and fibrosis via transforming growth factor-β (TGF-β) and correlates with fibrosis indices such as FIB-4 [15,16]. Conversely, miR-197-3p (miR-197) is typically downregulated in advanced MAFLD and demonstrates an inverse relationship with inflammatory markers [17,18], while miR-99a-5p (miR-99a) regulates mechanistic target of rapamycin (mTOR) and insulin-like growth factor 1 receptor (IGF1R), contributing to the suppression of lipogenesis and inflammation [19,20]. Collectively, these findings highlight the role of selected miRNAs in the pathogenesis and phenotypic variation of MAFLD subtypes.

Despite growing evidence supporting the involvement of miRNAs in fatty liver disease, their ability to distinguish among distinct MAFLD phenotypes remains poorly understood, particularly in relation to the biological heterogeneity of T2D-MAFLD, OB-MAFLD, and L-MAFLD. Our previous research demonstrated significant metabolic and inflammatory differences across these phenotypes [21], highlighting the need for reliable molecular markers for their early and non-invasive differentiation. Therefore, this study aimed to characterize the expression profiles of metabolic-related miRNAs in patients with MAFLD compared with healthy controls and to evaluate whether phenotype-specific expression patterns could independently discriminate MAFLD subtypes. Furthermore, we assessed whether integrating the candidate miRNAs with conventional serum biomarkers could improve diagnostic accuracy and enable precise phenotypic stratification, thereby supporting their potential clinical utility as adjunctive tools for early detection and personalized disease management.

## 2. Materials and Methods

### 2.1. Study Design and Setting

This comparative observational study was conducted at the Pakistan Aeronautical Complex Hospital (PACH), Kamra, in collaboration with Armed Forces Institute of Pathology (AFIP), National University of Medical Sciences, Rawalpindi, Pakistan, between November 2021 and March 2024. The study protocol received approval from the Institutional Review Boards of PACH and AFIP. All patients provided written informed consent, consistent with the ethical guidelines of the Declaration of Helsinki (2013).

### 2.2. Patient Selection

A total of 393 patients, aged 20–70 years, were selected based on a fatty liver index (FLI) ≥ 30 [22], ultrasound-proven hepatic steatosis, and the presence of either type 2 diabetes mellitus (T2D; fasting plasma glucose ≥ 7.0 mmol/L or hemoglobin A1C ≥ 6.5%), obesity (body mass index [BMI] ≥ 23 kg/m^2^), or BMI < 23 kg/m^2^ in lean patients [2]. Healthy controls (*n* = 109) exhibited no evidence of liver or metabolic dysfunction. Exclusion criteria included viral hepatitis, alcohol use, hepatotoxic drugs, pregnancy, or autoimmune liver disease.

### 2.3. Clinical Assessment and Biochemical Analysis

Clinical and anthropometric data were collected at the time of enrollment by trained medical staff using standardized forms. Medical history and a physical examination were conducted in the medical outpatient department of PACH, Kamra, Pakistan. Anthropometric measurements including weight, waist circumference, and blood pressure were recorded.

Venous blood samples (10 mL) were collected following a 10–12 h overnight fast. Fasting plasma glucose (FPG), lipid profile, liver enzymes, and high-sensitivity C-reactive protein (hs-CRP) were analyzed using an automated chemistry analyzer (Cobas^®^ c501, Roche Diagnostics, Basel, Switzerland). Serum insulin levels were measured using a chemiluminescent immunoassay on the Cobas^®^ e411 platform (Roche Diagnostics, Basel, Switzerland). Platelet counts were determined using an automated hematology analyzer (Sysmex^®^ XP-100, Sysmex Corporation, Kobe, Japan).

Inflammatory and metabolic markers including interleukin-6 (IL-6), tumor necrosis factor-alpha (TNF-α), leptin, adiponectin, malondialdehyde (MDA), and CK-18 were quantified using enzyme-linked immunosorbent assay kits following the manufacturers’ instructions (Elabscience^®^, Houston, TX, USA). All assays were performed in duplicate, and quality controls were within established limits.

Hepatic steatosis was confirmed via B-mode ultrasonography performed by an experienced radiologist using standardized diagnostic criteria, including hepato-renal echo contrast, increased hepatic echogenicity, reduced posterior beam penetration, and obscured vascular architecture. A second radiologist independently confirmed all findings, blinded to the initial assessment and laboratory results. Detailed clinical and biochemical methodologies have been previously described [21].

### 2.4. Selection of Target miRNAs

In this study, we employed a candidate-based approach to evaluate circulating miRNAs associated with distinct phenotypic subtypes of MAFLD. Candidate miRNAs were selected based on prior literature search, and a panel of eight miRNAs most relevant to MAFLD pathogenesis was selected [6,7,10,11]. The selected miRNAs were miR-122, miR-103a, miR-222, miR-15a, miR-34a, miR-192, miR-197, and miR-99a. The sequences of these miRNAs, used to design the primers, are listed in Table S1 (Supplementary Materials).

### 2.5. Quantification of miRNAs

#### 2.5.1. Blood Collection and Storage

Plasma blood was drawn into EDTA tubes (5 mL) and separated by centrifugation at 4000 rpm for 10 min at 4 °C within 1 h. Subsequently, 250 µL aliquots were mixed with 750 µL of TRIzol™ LS Reagent (Thermo Fisher Scientific, Carlsbad, CA, USA) and stored at −80 °C.

#### 2.5.2. RNA Extraction

RNA was extracted using the TRIzol™ LS protocol [23], with 3.5 µL of synthetic cel-miR-39-3p (Qiagen, Hilden, Germany) added as an external control. RNA purity and concentration were assessed via NanoDrop spectrophotometry (Thermo Fisher Scientific Inc., Wilmington, DE, USA), (A260/A280: 1.8–2.1), and samples were stored at −80 °C.

#### 2.5.3. Quantitative Real-Time PCR (RT-qPCR)

c-DNA synthesis was performed using the miRNA All-In-One cDNA Synthesis Kit (Applied Biological Materials Inc., Richmond, BC, Canada) with 300 ng of total RNA per reaction. PCR was performed using Bright Green Master Mix with specific primers on a Rotor-Gene™ Q thermocycler (Qiagen, Hilden, Germany). The cycling conditions included an initial denaturation at 95 °C for 10 min, followed by 40 cycles of 95 °C for 10 s, 63 °C for 15 s, and 72 °C for 10 s. Ct values > 35 were excluded. Each sample was run in duplicate, including a control.

miRNA expression was normalized using the ΔCt method, calculated as ΔCt = Ct (target miRNA) − Ct (cel-miR-39-3p). Linear expression was then calculated using the following formula: 2^−ΔCt^ [24].

### 2.6. Sample Size and Statistical Analysis

Sample size was estimated using G*Power (version 3.1.9.2, Heinrich Heine University Düsseldorf, Germany), assuming an effect size of 0.2, 95% power, and α = 0.05, which yielded 390 participants (130 per phenotype) according to Cohen’s criteria [25]. Data were analyzed using SPSS (IBM SPSS Statistics for Windows, Version 21.0. Armonk, NY, USA). Normality of the data was assessed using the Shapiro–Wilk test. Continuous variables are presented as the median (inter-quartile range, IQR). The Mann–Whitney U test was used for comparisons between MAFLD and control groups, while Kruskal–Wallis with post hoc analysis was employed for comparisons between phenotypes.

Phenotype discrimination was assessed using univariable and multivariable multinomial logistic regression, comparing T2D-MAFLD, OB-MAFLD, and L-MAFLD. Internal validation was performed via non-parametric bootstrapping with 1000 resamples, and bootstrap-adjusted *p* values were employed to identify robust predictors. Following coefficient validation, predicted probabilities from the final multinomial models were utilized to generate receiver operator characteristic (ROC) curves evaluating combined panels of miRNAs and biomarkers for differentiating each phenotype. Diagnostic performance was assessed by calculating the area under the receiver operating characteristic curve (AUC) for individual microRNAs, as well as for phenotype-specific combined models constructed from significant predictors and for the composite significant microRNA panel. Optimal cut-off values for each phenotype were determined using the Youden Index to maximize sensitivity and specificity; statistical significance was established at *p* < 0.05.

## 3. Results

### 3.1. Patient Characteristics

**Figure 1:**
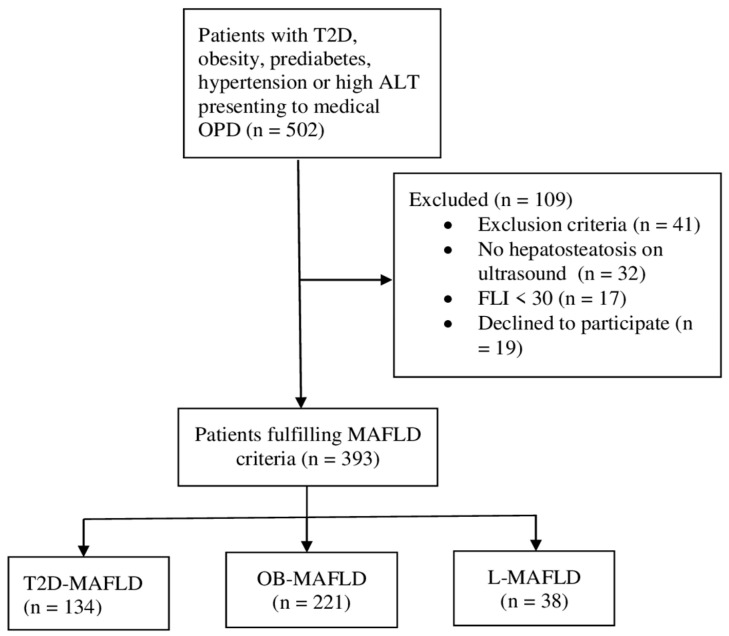
Flow diagram of the patient selection process. T2D: type 2 diabetes mellitus; ALT: alanine aminotransferase; OPD: out-patient department; FLI: fatty liver index; MAFLD: metabolic dysfunction-associated fatty liver disease; OB: obese; L: lean.

The patient selection process is summarized in Figure 1. A total of 393 patients meeting the MAFLD diagnostic criteria were categorized into three phenotypes: T2D-MAFLD (*n* = 134, 34.0%), OB-MAFLD (*n* = 221, 56.0%), and L-MAFLD (*n* = 38, 10.0%). Despite extended sampling over 2.5 years, the required number of lean cases could not be achieved due to the low regional prevalence of L-MAFLD (~3.0%) [26].

### 3.2. Differential Expression of Circulating miRNAs in MAFLD Patients and Controls

Compared with healthy controls, patients with MAFLD exhibited significantly altered expression of most circulating miRNAs (Table 1). ΔCt analysis revealed that miR-122, miR-103a, miR-222, miR-15a, and miR-34a were significantly upregulated in MAFLD (all *p* < 0.05). Conversely, miR-197 and miR-99a were downregulated in MAFLD (* *p* < 0.001 for both). Expression of miR-192 did not differ significantly between patients and controls (*p* = 0.477). These findings suggest a predominantly upregulated miRNA signature in MAFLD, with the exception of miR-197 and miR-99a.

### 3.3. Phenotype-Specific miRNA Expression Across MAFLD Subgroups

Phenotype-specific patterns were observed when comparing T2D-MAFLD, OB-MAFLD, and L-MAFLD (Table 2). miR-122 and miR-222 exhibited the greatest upregulation in the OB phenotype, whereas miR-103a and miR-34a displayed maximal expression in the T2D phenotype (all *p* < 0.001). miR-15a was significantly elevated in lean individuals compared to both the OB and T2D phenotypes (*p* < 0.001). Conversely, miR-192 expression was reduced in lean subjects relative to the T2D and OB phenotypes (*p* < 0.001). miR-197 expression was suppressed in the T2D phenotype (*p* < 0.001), and miR-99a exhibited the greatest downregulation in the OB subgroup (*p* < 0.001) for the comparison between T2D and OB, and between OB and lean.

### 3.4. Multinomial Logistic Regression Based on Expression of miRNAs Across MAFLD Phenotypes

Age-adjusted multinomial regression revealed distinct phenotype-specific miRNA expression patterns across MAFLD subtypes (Table 3 and Table S2). Higher expression of miR-103a and miR-34a was strongly associated with T2D-MAFLD, increasing its odds by approximately 2.7–3.0-fold relative to L-MAFLD, while downregulation of miR-197 independently increased the likelihood of T2D-MAFLD by nearly twofold compared with both OB-MAFLD and L-MAFLD. OB-MAFLD exhibited elevated expression of miR-122, miR-103a, and miR-222, conferring odds ratios 1.5–2.3 higher than those observed in L-MAFLD. Notably, downregulation of miR-99a demonstrated the strongest association with obesity, increasing the likelihood of OB-MAFLD by approximately 4.2-fold compared to L-MAFLD and by 4.7-fold compared to T2D-MAFLD. In contrast, higher miR-15a expression independently favored L-MAFLD, increasing its odds by approximately 1.6–1.7-fold relative to both OB- and T2D-MAFLD.

In summary, miR-103a and miR-34a were the most robust discriminators of the T2D phenotype, miR-122 and miR-222 characterized the OB phenotype, and miR-15a was strongly associated with the lean phenotype. miR-197 and miR-99a provided inverse or phenotype-specific signals, with miR-197 linked to T2D and miR-99a strongly defining OB-MAFLD.

### 3.5. Diagnostic Performance of Individual and Combined miRNA Signatures

ROC analysis revealed distinct phenotype-specific discriminatory patterns for individual miRNAs (Table 4). In T2D-MAFLD, miR-103 (AUC 0.727), miR-34a (AUC 0.699), and miR-197 (AUC 0.784) exhibited the strongest individual performance, while in OB-MAFLD, miR-99a demonstrated excellent accuracy (AUC 0.869), followed by miR-122 (AUC 0.652) and miR-222 (AUC 0.666). L-MAFLD was uniquely characterized by a high AUC for miR-15a (0.776), with all other markers performing poorly (AUC < 0.5).

Optimized phenotype-specific combined miRNA panels, selected based on logistic regression and moderate diagnostic accuracy within each phenotype (mir-103a, micR-34a, and miRN-197 for T2D-MAFLD, miR-122, miR-222, and miR-99a for OB-MAFLD, and miR-15a and miR-197 for L-MAFLD), were assessed for combined panel performance. The combination of miRNAs substantially improved diagnostic accuracy across all phenotypes, resulting in areas under the receiver operating characteristic curve (AUCs) of 0.805 for T2D-MAFLD, 0.876 for OB-MAFLD, and 0.786 for L-MAFLD. These combined models consistently outperformed individual miRNAs and demonstrated the highest overall discriminatory ability.

### 3.6. Diagnostic Enhancement of Biomarker Models Through miRNA Integration

**Figure 2:**
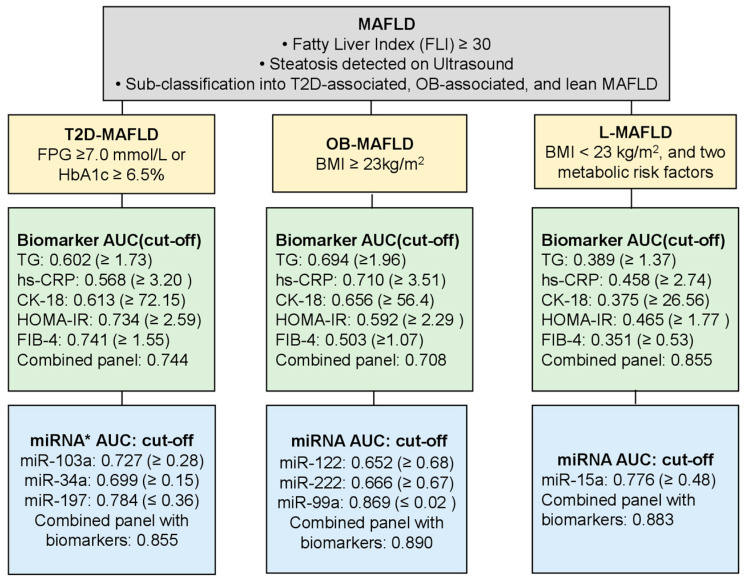
A stepwise diagnostic framework integrating clinical criteria, biochemical markers, and circulating miRNAs for MAFLD phenotype stratification. MAFLD, metabolic dysfunction-associated fatty liver disease; FLI, fatty liver index; BMI, body mass index; T2D, type 2 diabetes mellitus; OB-MAFLD, obesity-associated MAFLD; L-MAFLD, lean associated MAFLD; TG, triglyceride; hs-CRP, high-sensitivity C-reactive protein; CK-18, cytokeratin-18; HOMA-IR, Homeostasis Model Assessment of Insulin Resistance; FIB-4, fibrosis-4 index; AUC, area under the receiver operating characteristic curve; miRNA, microRNA. * ΔCt, delta cycle threshold by RT-qPCR, real-time quantitative polymerase chain reaction. Cut-off values for biomarkers were derived from a previous study.

Based on the integrated analysis, we formulated a stepwise diagnostic scheme illustrating how biochemical markers and phenotype-specific circulating miRNAs may be combined to refine MAFLD subtype classification. The scheme incorporates initial steatosis confirmation, baseline clinical phenotyping, biochemical risk assessment, and targeted microRNA profiling, followed by integrated modeling using predicted probabilities (Figure 2).

For T2D-MAFLD, the biomarker-only model (AUC 0.744; 95% CI 0.693–0.796) exhibited good discrimination, which substantially improved upon the addition of miR-103, miR-34a, and miR-197 (AUC 0.855; 95% CI 0.817–0.893). A similar improvement was observed in OB-MAFLD, where the inclusion of miR-122, miR-222, and miR-99a increased the AUC from 0.708 (95% CI 0.657–0.759) to 0.890 (95% CI 0.856–0.923). Notably, the biomarker-only model discriminated L-MAFLD (AUC 0.855; *p* < 0.001), while incorporation of miR-15a produced a strong diagnostic signal (AUC 0.883; 95% CI 0.833–0.932). This approach reflects the observed improvement in diagnostic discrimination when microRNAs were added to conventional biomarkers across all phenotypes.

### 3.7. Clinical Diagnostic Utility of Individual miRNAs

ROC-derived diagnostic parameters indicated that individual miRNAs exhibited phenotype-specific patterns of clinical utility in patients with MAFLD (Table 5). In the T2D-MAFLD cohort, miR-197-3p demonstrated the highest overall diagnostic performance, achieving balanced sensitivity (81%) and specificity (80%), alongside strong positive predictive value (PPV 67.9%) and negative predictive value (NPV 88.9%). miR-103a also performed moderately well (sensitivity and specificity ~75%), while most other miRNAs exhibited high sensitivity but low specificity, thereby limiting their standalone clinical applicability.

In OB-MAFLD, miR-99a exhibited excellent specificity (97.5%) and moderate positive predictive value (PPV; 57.2%), suggesting its potential as a confirmatory biomarker for obesity-related MAFLD. miR-122 and miR-222 offered more balanced discrimination (sensitivities and specificities of approximately 65–67%), supporting their use as first-line screening markers.

L-MAFLD showed a distinct signature, with miR-15 achieving high specificity (87.0%) and excellent NPV (95.7%), making it clinically valuable for ruling out lean disease. miR-197 and miR-99a also demonstrated high sensitivity (>94%) but low specificity, suggesting utility as preliminary screening indicators.

Overall, these findings demonstrate that while no single miRNA provides universal diagnostic performance across all phenotypes, specific miRNAs offer clinically meaningful predictive value within individual MAFLD subtypes. High-specificity markers (e.g., miR-99a in OB-MAFLD and miR-15a in L-MAFLD) may be particularly valuable in confirmatory diagnostic pathways, whereas high-sensitivity markers (e.g., miR-197 in T2D-MAFLD) may serve as effective screening tools. Integration of such phenotype-tailored miRNAs could enhance early, non-invasive disease stratification in clinical practice.

## 4. Discussion

This study elucidates the molecular differences among the three phenotypes of MAFLD through selected miRNA profiling and assesses their diagnostic performance alongside metabolic, inflammatory, and fibrotic parameters. Of the miRNAs studied in MAFLD, six—miR-122, -103a, -222, -15a, -34a, and -192—were upregulated, while two miRNAs—miR-197 and -99a—were downregulated, as compared to healthy controls. Previous research has demonstrated variable miRNA expression in fatty liver, hepatic steatosis, and fibrosis, owing to their role in post-transcriptional gene regulation [14,27].

miR-122 is the most abundant miRNA in the liver; studies have consistently reported its upregulation in fatty liver [20,28]. In this study, miR-122 was found to be upregulated across all subgroups, with the highest expression observed in obese patients with MAFLD, which aligns with its role in hepatic lipid metabolism and adipogenesis [29]. Inhibition of miR-122 reduces circulating cholesterol, downregulates lipogenic pathways, and enhances fatty acid oxidation. In glucose metabolism, miR-122 indirectly modulates gluconeogenesis and insulin sensitivity through AMPK signaling and glucose-6-phosphatase regulation [30]. However, some studies suggest a decline in miR-122 with advanced fibrosis, indicating that its diagnostic value may be stage-dependent [15]. miR-103a exhibited the highest expression in patients with type 2 diabetes and MAFLD, demonstrating significant differences between all phenotypes, consistent with its role in promoting insulin resistance via suppression of caveolae-associated vascular protein 1 (CAV-1) and upregulation of secreted frizzled-related protein-4 (SFRP4) [10,31]. Experimental suppression of miR-103a has been shown to improve lipid oxidation, reduce oxidative stress, and ameliorate histological steatosis, further supporting its pathogenic role [32].

miR-222 was elevated in T2D- and OB-MAFLD but markedly suppressed in L-MAFLD. This pattern reflects its established involvement in adipogenesis and impaired lipid oxidation through the targeting of B-cell translocation gene 2 (BTG2), adiponectin receptor-1 (AdipoR1), and acyl-CoA oxidase-1 (ACOX1) [7,8,9]. Previous studies in obese children have shown that combined elevation of miR-122 and miR-222 is associated with an increased risk of obesity and adverse metabolic profiles [33,34].

In contrast, miR-15a was most highly expressed in L-MAFLD, with sequentially lower levels in OB and T2D phenotypes. This observation aligns with previous reports of its elevation in metabolic syndrome [11] and reduced expression in obese versus lean individuals [34].

miR-34a was significantly upregulated in T2D- and OB-MAFLD, consistent with prior evidence linking miR-34a to peroxisome proliferator-activated receptor-alpha (PPARα) suppression, inflammation, and hepatocyte apoptosis [13]. miR-192 has been linked to fibrosis progression via TGF-β signaling [35], but in this study, it was found to be insignificantly elevated in the MAFLD group compared to controls. In contrast, miR-197 and miR-99a were downregulated, particularly in metabolically severe phenotypes (T2D and OB). These trends align with earlier findings demonstrating reduced expression of these miRNAs in fatty liver and advanced fibrosis, suggesting a potential protective or anti-inflammatory role [19].

The ability of the miRNAs to accurately predict MAFLD better and earlier has made them a topic of interest in the field of medicine [18]. The miRNAs in our cohort also demonstrated strong predictability toward each phenotype. High expression of miR-122 is associated with higher odds of having OB-MAFLD, and miR-103a and miR-34a were strongest in predicting T2D-MAFLD, while miR-15a was most predictive of L-MAFLD, suggesting its specificity for non-obese, non-diabetic hepatic steatosis. Among downregulated miRNAs, miR-197 strongly predicted T2D-MAFLD, while miR-99a was most strongly associated with OB-MAFLD, confirming their potential as negative phenotype markers. These findings support the utility of circulating miRNAs as a non-invasive, quantitative tool for steatosis estimation, potentially complementing or replacing imaging and biopsy in MAFLD stratification [36,37].

MiRNAs have repeatedly demonstrated promising potential for the diagnosis, staging, and monitoring of fatty liver [38]. However, individual miRNAs have exhibited variable diagnostic accuracies across different stages of the disease. Combinations of miRNAs have generally shown improved diagnostic accuracy compared to individual miRNAs. Stoica et al. reported that different combinations of miRNAs showed different AUCs; for example, combining miR-122 and miR-33 improved the AUC to 0.86 [15]. This study’s cohort also displayed diagnostic accuracy variation across phenotypes. In T2D-MAFLD, miR-197 and miR-103a were the most accurate individual markers, comparable to previously reported accuracies in steatohepatitis [17]. Nevertheless, the addition of selected miRNAs (miR-103a, miR-34, and miR-197) from regression analysis further enhanced the AUC values. Similarly, in OB-MAFLD, miR-99a demonstrated good diagnostic accuracy, which was further improved by adding significant miRNAs (miR-122 and miR-222), consistent with the findings of Kim et al., who showed that a combined panel of eight miRNAs was more promising for diagnosing steatohepatitis [39]. In L-MAFLD, the diagnostic performance of individual miRNAs was generally poor, except for miR-15a (AUC 0.776), which did not substantially improve after adding miR-197. This observation represents a significant finding for patients who are challenging to diagnose during routine medical checkups.

In our recent publication, we reported the association of various biomarkers with each phenotype of MAFLD [21]. The biomarkers that correlated with the pathological pathways of the selected miRNAs were selected; for example, TG is the marker of lipogenesis [40], hs-CRP is for detecting systemic inflammation [41], CK-18 reflects the burden of apoptosis [42], HOMA-IR is the surrogate marker for insulin resistance [43], and FIB-4 is the indirect marker of fibrosis [44]. These biomarkers were then analyzed as a combined panel to determine their discriminatory ability for each phenotype. It is noteworthy that the panel showed marked discrimination for L-MAFLD. Subsequently, we integrated the miRNAs that had shown good diagnostic accuracies in each phenotype into the biomarker panel, with the aim of improving the diagnostic performance of each panel. The results of these combined panels were promising, with AUC values showing substantial improvement to 0.855 (T2D), 0.890 (OB), and 0.883 (lean), as reported in earlier work [44]. These results demonstrate the added value of miRNA panels in enhancing the discriminatory capacity of existing clinical markers, particularly in diagnostically challenging subtypes such as L-MAFLD [45].

The incorporation of circulating miRNA profiling into current diagnostic workflows may improve early stratification of MAFLD phenotypes, complementing biochemical markers and imaging-based approaches. Given the increasing availability and decreasing cost of qPCR-based assays, miRNA testing may offer a feasible and minimally invasive adjunct for risk stratification in routine hepatology practice.

While the present findings provide novel insights into phenotype-specific circulating miRNA signatures in MAFLD, several considerations merit discussion. First, histological confirmation was not performed, and MAFLD diagnosis and phenotypic classification relied on validated non-invasive criteria. Second, the relatively smaller representation of L-MAFLD reflects its lower prevalence in the regional population and may limit statistical power for this subgroup; however, robust internal validation was applied to mitigate this constraint. Third, the single-center design may restrict generalizability across diverse ethnic and metabolic populations, underscoring the need for external validation in multi-center cohorts. Finally, the cross-sectional nature of the study precludes causal inference, and further functional investigation is warranted to elucidate mechanistic pathways linking miRNA dysregulation to MAFLD phenotypes.

## 5. Conclusions

This study demonstrates that distinct circulating miRNA signatures provide phenotype-specific discrimination of MAFLD subtypes. miR-103, miR-34a, and miR-197 were characteristic of T2D-MAFLD, and miR-122, miR-222, and miR-99a defined OB-MAFLD, while miR-15a specifically indicated L-MAFLD. ROC analysis confirmed their independent and phenotype-specific diagnostic accuracy. Integration of multi-microRNA panels with conventional biochemical markers significantly enhanced diagnostic accuracy, particularly for L-MAFLD, supporting their potential as non-invasive tools for early MAFLD stratification and warranting validation in larger, multi-center cohorts.

## Figures and Tables

**Table 1:** Comparison of demographics and miRNA expression between control and MAFLD groups.

Parameter	Controls (*n* = 109)	MAFLD (*n* = 393)	* *p*
Sex			
Female	48 (44.0) ^†^	152 (38.7)	0.32
Male	61 (56.0)	241 (61.3)	
Age (years)	43.32 ± 8.36 ^‡^	44.77 ± 8.07	0.10
hsa-miR-122-5p	3.05 (1.64) ^§^	1.45 (1.44)	<0.001
hsa-miR-103a-3p	3.57 (1.30)	2.09 (1.30)	<0.001
hsa-miR-222-3p	3.03 (1.07)	1.50 (1.15)	<0.001
hsa-miR-15a-5p	2.12 (1.29)	1.89 (1.08)	0.018
hsa-miR-34a-5p	4.13 (1.22)	3.01 (1.72)	<0.001
hsa-miR-192-5p	2.08 (1.30)	1.88 (1.28)	0.477
hsa-miR-197-3p	0.81 (1.16)	1.26 (1.15)	<0.001
hsa-miR-99a-5p	0.93 (1.35)	1.71 (1.25)	<0.001

MAFLD: metabolic dysfunction-associated fatty liver disease; hsa: Homo sapiens; miR: microRNA. microRNA data are presented as ∆Ct, where Ct: cycle threshold; ∆Ct: Ct miR minus Ct miR-Cel-39-3p. Lower ∆Ct values indicate higher expression levels, and vice versa. * Between groups by independent *t*-test, Mann–Whitney or chi-square test as appropriate. ^†^ Values are frequency with percentage. ^‡^ Values are mean ± standard deviation (SD). ^§^ Values are median ∆Ct with inter-quartile range (IQR).

**Table 2:** Comparison of miRNA expression among the three phenotypes of MAFLD.

miRNA	T2D (*n* = 134)	Obese (*n* = 221)	Lean (*n* = 38)	*p* ^1^	*p* ^2^	*p* ^3^
hsa-miR-122-5p	1.95 (1.23) ^4^	1.24 (1.31)	2.55 (1.41)	<0.001	0.002	<0.001
hsa-miR-103a-3p	1.29 (0.92)	2.22 (1.07)	3.08 (1.91)	<0.001	<0.001	<0.001
hsa-miR-222-3p	1.91(1.01)	1.27 (0.82)	2.90 (1.45)	<0.001	<0.001	<0.001
hsa-miR-15-5p	2.07 (1.34)	1.82 (1.00)	0.97 (0.80)	0.077	<0.001	0.001
hsa-miR-34a-5p	2.42 (1.17)	3.12 (1.64)	3.96 (1.39)	<0.001	<0.001	<0.001
hsa-miR-192-5p	1.72 (1.56)	1.87 (1.06)	2.88 (1.48)	0.625	<0.001	<0.001
hsa-miR-197-3p	2.02 (0.83)	1.08 (0.52)	0.90 (0.53)	<0.001	<0.001	0.134
hsa-miR-99a-5p	0.875 (0.45)	1.99 (0.52)	0.78 (0.33)	<0.001	0.178	<0.001

MAFLD: metabolic dysfunction-associated fatty liver disease; T2D: type 2 diabetes mellitus; hsa: Homo sapiens; miR: microRNA. ^1^ Between T2D and obese groups. ^2^ Between T2D and lean groups. ^3^ Between obese and lean groups. ^4^ Values are median ∆Ct with inter-quartile range (IQR). All *p* values are from Mann–Whitney U test.

**Table 3:** Multinomial regression analysis of miRNA expression with the three MAFLD phenotypes.

miRNA	Comparison	B	SE	Exp (B)	95% CI for Exp (B)	*p* ^1^	Multi-Boot-*p* ^2^
hsa-miR-122-5p	T2D vs. OB	0.083	0.069	1.087	0.950–1.243	0.225	0.214
	OB vs. Lean	−0.387	0.129	0.679	0.528–0.875	0.003	0.003
	T2D vs. Lean	−0.303	0.140	0.738	0.562–0.971	0.030	0.038
hsa-miR-103a-3p	T2D vs. OB	−0.344	0.102	0.709	0.580–0.866	0.001	0.002
	OB vs. Lean	−0.662	0.207	0.516	0.344–0.774	0.001	0.003
	T2D vs. Lean	−1.007	0.221	0.365	0.237–0.564	<0.001	0.001
hsa-miR-222-3p	T2D vs. OB	0.069	0.100	1.071	0.880–1.304	0.492	0.409
	OB vs. Lean	−0.830	0.203	0.436	0.293–0.649	<0.001	0.001
	T2D vs. Lean	−0.761	0.211	0.467	0.309–0.706	<0.001	0.001
hsa-miR-15a-5p	T2D vs. OB	0.059	0.119	1.061	0.840–1.341	0.619	0.686
	OB vs. Lean	0.472	0.195	1.603	1.094–2.348	0.015	0.016
	T2D vs. Lean	0.531	0.208	1.701	1.131–2.558	0.011	0.011
hsa-miR-34a-5p	T2D vs. OB	−0.393	0.132	0.675	0.521–0.873	0.003	0.004
	OB vs. Lean	−0.700	0.257	0.467	0.337–0.648	0.007	0.010
	T2D vs. Lean	−1.093	0.274	0.335	0.196–0.573	<0.001	0.001
hsa-miR-192-5p	T2D vs. OB	0.133	0.105	1.142	0.929–1.403	0.207	0.187
	OB vs. Lean	−0.497	0.199	0.609	0.412–0.898	0.012	0.010
	T2D vs. Lean	−0.364	0.207	0.695	0.463–1.043	0.079	0.083
hsa-miR-197-3p	T2D vs. OB	0.662	0.153	1.940	1.436–2.620	<0.001	0.001
	OB vs. Lean	0.109	0.247	1.115	0.687–1.810	0.660	0.637
	T2D vs. Lean	0.771	0.269	2.162	1.277–3.660	0.004	0.009
hsa-miR-99a-5p	T2D vs. OB	−1.550	0.217	0.212	0.139–0.325	<0.001	0.001
	OB vs. Lean	1.434	0.318	4.196	2.250–7.825	<0.001	0.004
	T2D vs. Lean	−0.116	0.311	0.891	0.484–1.640	0.710	0.793

MAFLD: metabolic dysfunction-associated fatty liver disease; hsa: Homo sapiens; miR: microRNA; OB: obese; T2D: type 2 diabetes mellitus; B: beta coefficient; SE: standard error; Exp (B): odds ratio; CI: confidence interval; multi-boot-*p*: multivariate bootstrapped *p* value. ^1^ *p* value derived from multivariate multinomial analysis of eight miRNAs within each phenotype versus other two phenotypes of MAFLD; ^2^ *p* value derived from multivariate analysis of eight miRNA panel within each phenotype versus other two phenotypes of MAFLD after bootstrapping.

**Table 4:** Discriminatory ability of single versus combined miRNAs in differentiating MAFLD phenotypes.

miRNA	T2D-MAFLD vs. Others AUC (95% CI) *p*	OB-MAFLD vs. Others AUC (95% CI) *p*	L-MAFLD vs. Others AUC (95% CI) *p*
hsa-miR-122-5p	0.415 (0.355–0.475) (0.006)	0.652 (0.595–0.709) (<0.001)	0.291 (0.200–0.382) (<0.001)
hsa-miR-103-3p	0.727 (0.673–0.782) (<0.001)	0.378 (0.320–0.437) (<0.001)	0.259 (0.169–0.349) (<0.001)
hsa-miR-222-3p	0.433 (0.373–0.492) (0.029)	0.666 (0.611–0.722) (<0.001)	0.204 (0.140–0.268) (<0.001)
hsa-miR-15-5p	0.411 (0.352–0.470) (0.004)	0.483 (0.425–0.542) (0.571)	0.776 (0.694–0.859) (<0.001)
hsa-miR-34a-5p	0.699 (0.646–0.753) (<0.001)	0.413 (0.356–0.470) (0.003)	0.233 (0.159–0.307) (<0.001)
hsa-miR-192-5p	0.547 (0.486–0.609) (0.126)	0.541 (0.483–0.600) (0.159)	0.262 (0.190–0.334) (<0.001)
hsa-miR-197-3p	0.784 (0.729–0.839) (<0.001)	0.296 (0.240–0.352) (<0.001)	0.344 (0.257–0.430) (0.002)
hsa-miR-99a-5p	0.191 (0.147–0.235) (<0.001)	0.869 (0.831–0.908) (<0.001)	0.255 (0.173–0.336) (<0.001)
Combined panel (significant miRNAs only) ^1^	0.805 (0.758–0.852) (<0.001)	0.876 (0.835–0.913) (<0.001)	0.787 (0.710–0.863) (<0.001)

MAFLD: metabolic dysfunction-associated fatty liver disease; AUC: area under the curve; hsa: Homo sapiens; miR: microRNA; OB: obese; T2D: type 2 diabetes mellitus; L: lean; CI: confidence interval; *p*: *p* value. ^1^ Optimized model based on logistic regression analysis.

**Table 5:** Clinical diagnostic utility of miRNAs across MAFLD phenotypes: ROC-derived cut-offs and predictive value.

	T2D-MAFLD vs. Others					OB-MAFLD vs. Others					L-MAFLD vs. Others				
	Cut-Off	Sen%	Spec%	PPV	NPV	Cut-Off	Sen%	Spec%	PPV	NPV	Cut-Off	Sen%	Spec%	PPV	NPV
miR-122-5p	0.0358	0.978	0.062	35	84.49	0.3481	0.683	0.663	72.23	61.98	0.0274	0.921	0.034	9.29	80.03
miR-103a-3p	0.2892	0.746	0.741	59.85	84.93	0.1351	0.846	0.209	57.85	51.4	0.2892	0.132	0.544	3.02	85.37
miR-222-3p	0.1028	0.91	0.162	35.98	77.67	0.3409	0.674	0.674	72.62	61.71	0.0304	0.974	0.023	9.67	89.17
miR-15a-5p	0.0774	0.91	0.12	34.86	72.04	0.2491	0.597	0.477	59.43	47.98	0.4763	0.632	0.87	34.31	95.65
miR-34a-5p	0.1502	0.604	0.71	51.87	77.6	0.0404	0.946	0.07	56.62	50.26	0.0476	0.711	0.062	7.53	66.63
miR-192-5p	0.0758	0.896	0.127	34.69	70.24	0.1901	0.814	0.343	61.39	58.97	0.0503	0.947	0.037	9.55	86.66
miR-197-3p	0.3566	0.806	0.803	67.92	88.89	1.8662	0.974	0.048	56.76	59	1.3153	0.947	0.068	9.84	92.27
miR-99a-5p	1.2306	0.985	0.039	34.66	83.4	0.0544	0.026	0.975	57.16	43.83	0.6938	0.842	0.087	9.01	83.68

T2D: type 2 diabetes; OB: obese; L: lean; Sen: sensitivity; Spec: specificity; PPV: positive predictive value; NPV: negative predictive value; miR: microRNA; MAFLD: metabolic dysfunction-associated fatty liver disease. Cut-offs were determined using ROC-derived thresholds based on 2^−ΔCt^ values.

## Data Availability

The raw data supporting the conclusions of this article will be made available by the authors on request.

## Supplementary Materials

The supporting information can be downloaded at: https://www.mdpi.com/article/10.3390/cimb48030272/s1.
