# Supplementary material for: Circulating MicroRNA Profiling for Phenotypic Stratification in Patients with Metabolic Dysfunction-Associated Fatty Liver Disease: A Candidate-Based Study
Source: Curr Issues Mol Biol. 2026 Mar 4;48(3):272. doi: 10.3390/cimb48030272 (PMC13024945; doi:10.3390/cimb48030272)
Supplement: Supplementary file 1 [file cimb-48-00272-s001.zip › cimb-4147150-supplementary.pdf]

Table S1: Sequences of tested miRNAs and spike-in reference used by the manufacturer to design primers for RT-qPCR

**miRNA/gene**	**miRBase Accession**	**Mature miRNA sequence**
hsa-mir-122-5p	MIMAT0000421	5’ UGGAGUGUGACAAUGGUGUUUG ’3
hsa-mir-103a-3p	MIMAT0000101	5’ AGCAGCAUUGUACAGGGCUAUGA’3
hsa-mir-222-3p	MIMAT0000279	5’ AGCUACAUCUGGCUACUGGGU ’3
hsa-mir-15a-5p	MIMAT0000068	5’ UAGCAGCACAUAAUGGUUUGUG ’3
hsa-mir-34a-5p	MIMAT0000255	5’ UGGCAGUGUCUUAGCUGGUUGU ’3
hsa-mir-192-5p	MIMAT0000222	5’ CUGACCUAUGAAUUGACAGCC’3
hsa-mir-197-3p	MIMAT0000227	5’ UUCACCACCUUCUCCACCCAGC ’3
hsa-mir-99a-5p	MIMAT0000097	5’ AACCCGUAGAUCCGAUCUUGUG’3
cel-miR-39-3p	MIMAT0000010	5’ UCACCGGGUGUAAAUCAGCUUG ’3

Table S2: Comparison of univariate multinomial regression analysis and multivariate after bootstrapping

miRNA	Univariable B	Univ OR	Univ 95% CI	Univ boot-p	Multivariable B	Multi OR	Multi 95% CI	Multi boot-p
**T2D vs OB**								
miR-122	0.098	1.103	1.001–1.216	0.050	0.083	1.087	0.950–1.243	0.214
miR-103	−0.304	0.738	0.638–0.853	0.001	−0.344	0.709	0.580–0.866	0.002
miR-222	0.131	1.140	0.981–1.326	0.106	0.069	1.071	0.880–1.304	0.409
miR-15a	0.057	1.059	0.895–1.253	0.513	0.059	1.061	0.840–1.341	0.686
miR-34a	−0.408	0.665	0.549–0.806	0.001	−0.393	0.675	0.521–0.873	0.004

miR-192	0.014	1.014	0.872–1.179	0.855	0.133	1.142	0.929–1.403	0.187
miR-197	0.865	2.374	1.830–3.081	0.001	0.662	1.940	1.436–2.620	0.001
miR-99a	−1.627	0.197	0.135–0.287	0.001	−1.550	0.212	0.139–0.325	0.001
**OB vs Lean**								
miR-122	−0.277	0.758	0.635–0.905	0.005	−0.387	0.679	0.528–0.875	0.003
miR-103	−0.501	0.606	0.473–0.777	0.001	−0.662	0.516	0.344–0.774	0.003
miR-222	−0.667	0.513	0.402–0.656	0.001	−0.830	0.436	0.293–0.649	0.001
miR-15a	0.582	1.789	1.376–2.325	0.001	0.472	1.603	1.094–2.348	0.016
miR-34a	−0.761	0.467	0.337–0.648	0.001	−0.700	0.467	0.337–0.648	0.010
miR-192	−0.585	0.557	0.423–0.734	0.001	−0.497	0.609	0.412–0.898	0.010
miR-197	0.078	1.082	0.800–1.463	0.521	0.109	1.115	0.687–1.810	0.637
miR-99a	1.739	5.694	3.510–9.237	0.001	1.434	4.196	2.250–7.825	0.004
**T2D vs Lean**								
miR-122	−0.179	0.836	0.697–1.004	0.059	−0.303	0.738	0.562–0.971	0.038
miR-103	−0.805	0.447	0.341–0.587	0.001	−1.007	0.365	0.237–0.564	0.001
miR-222	−0.535	0.585	0.457–0.750	0.001	−0.761	0.467	0.309–0.706	0.001
miR-15a	0.639	1.894	1.434–2.503	0.001	0.531	1.701	1.131–2.558	0.011
miR-34a	−1.169	0.311	0.217–0.445	0.001	−1.093	0.335	0.196–0.573	0.001
miR-192	−0.571	0.565	0.425–0.751	0.001	−0.364	0.695	0.463–1.043	0.083
miR-197	0.943	2.568	1.779–3.706	0.001	0.771	2.162	1.277–3.660	0.009
miR-99a	0.113	1.119	0.756–1.657	0.584	−0.116	0.891	0.484–1.640	0.793

miR: microRNA; OB: Obese; T2D: type 2 diabetes mellitus; B: beta coefficient; OR: odds ratio; Univ: univariable; boot: bootstrapped; CI: confidence interval; Multi : multivariable
